# Factors associated with misdiagnosis: the usefulness of necropsy

**DOI:** 10.1186/2197-425X-3-S1-A728

**Published:** 2015-10-01

**Authors:** G Moreno, P Perelló, L Claverias, J Leache, J Marín, V Blázquez, JJ Sirvent, A Rodríguez, M Bodí, M Magret

**Affiliations:** Hospital Universitari Joan XXIII de Tarragona, ICU, Tarragona, Spain; Hospital Universitari Joan XXIII de Tarragona, PD, Tarragona, Spain

## Introduction

In the past decades, autopsy rates have decreased over the time partly because of the availability of advanced and new technologies. However, autopsies remain a valuable examination as a tool for quality control as they help detecting clinical misdiagnosis. Patients in the intensive care unit may be especially susceptible to suffer harm from diagnostic errors. In the current literature discrepancies between clinical and pathological diagnoses are well documented, however we have not found studies that analyze the factors associated with the occurrence of such errors.

## Objectives

Identify factors associated with major misdiagnosis.

Analyze the differences between clinical and pathological diagnosis.

Examine the most common clinical diagnostic errors.

Determine if they remain present throughout time.

## Methods

This is an observational retrospective study over a period of 14 years. The comparisons between clinical and pathological diagnoses were classified as major and minor discrepancies or as a complete agreement according to the classification proposed by Goldman. The relation between Goldman/year of study was performed using Chi-square test of Pearson(χ2). To analyze factors associated with misdiagnosis a multivariate analysis was made using a multiple linear regression with diagnostic category as the dependent variable.

## Results

Of the total of 2076 patients who died in the 14-year period, 289(14%) had a post-mortem examination. The mean age was 62.3 years(SD 15.3), with a median APACHE II 22(IQR 11-33) and 67% were male. According to the Goldman classification, in most autopsies there was concordance between both diagnosis(64%), followed by major errors(30%). The most frequent major errors were infections(48%) followed by cardiovascular diseases(30%). A decrease on the number of major errors was not observed over time in the study period(χ2 61.3; p = 0.177). No relationship was found between length of stay and the major errors(p = 0.257). To assess factors associated with major errors we included as independent variables: age, APACHE II, length of ICU stay, immunosuppression, sepsis on admission and the diagnostic category (medical/surgical). Only the diagnostic category was associated with the occurrence of major errors (OR=3.04; 95% CI = 1.26 to 7.31; p = 0.013), being more frequent in medical patients.

## Conclusions

Medical patients have a higher risk of suffering major errors. In most autopsies there was concordance between the clinical and pathological diagnosis but in a third of cases major errors were detected. Infectious diseases were the most frequently unperceived. The major misdiagnosis remained present during the study period. Therefore, the persisting discordance between clinical and autopsy diagnoses is a good reason to continue performing autopsies.

